# Temporal consistency between gross primary production and solar-induced chlorophyll fluorescence in the ten most populous megacity areas over years

**DOI:** 10.1038/s41598-017-13783-5

**Published:** 2017-11-02

**Authors:** Yaoping Cui, Xiangming Xiao, Yao Zhang, Jinwei Dong, Yuanwei Qin, Russell B. Doughty, Geli Zhang, Jie Wang, Xiaocui Wu, Yaochen Qin, Shenghui Zhou, Joanna Joiner, Berrien Moore

**Affiliations:** 1 Collaborative Innovation Center for the “Three Modernization” Harmonious Development of Central Plains Economic Region, Laboratory of Geospatial Technology for the Middle and Lower Yellow River Regions, Henan University, Kaifeng, Henan 475004 China; 2 Department of Microbiology and Plant Biology, Center for Spatial Analysis, University of Oklahoma, Norman, Oklahoma 73072 USA; 3 Ministry of Education Key Laboratory of Biodiversity Science and Ecological Engineering, Institute of Biodiversity Science, Fudan University, Shanghai, 222200 China; 4 Institute of Geographic Sciences and Natural Resources Research, Chinese Academy of Sciences, Beijing, 100101 China; 5 NASA Goddard Space Flight Center, Greenbelt, MD 20771 USA; 6 College of Atmospheric and Geographic Sciences, University of Oklahoma, Norman, Oklahoma 73072 USA

## Abstract

The gross primary production (GPP) of vegetation in urban areas plays an important role in the study of urban ecology. It is difficult however, to accurately estimate GPP in urban areas, mostly due to the complexity of impervious land surfaces, buildings, vegetation, and management. Recently, we used the Vegetation Photosynthesis Model (VPM), climate data, and satellite images to estimate the GPP of terrestrial ecosystems including urban areas. Here, we report VPM-based GPP (GPP_vpm_) estimates for the world’s ten most populous megacities during 2000–2014. The seasonal dynamics of GPP_vpm_ during 2007–2014 in the ten megacities track well that of the solar-induced chlorophyll fluorescence (SIF) data from GOME-2 at 0.5° × 0.5° resolution. Annual GPP_vpm_ during 2000–2014 also shows substantial variation among the ten megacities, and year-to-year trends show increases, no change, and decreases. Urban expansion and vegetation collectively impact GPP variations in these megacities. The results of this study demonstrate the potential of a satellite-based vegetation photosynthesis model for diagnostic studies of GPP and the terrestrial carbon cycle in urban areas.

## Introduction

At present 54% of the world’s population resides in urban areas^1^. Most cities are experiencing an acceleration of urbanization in terms of both population growth and area expansion^1,2^. Although urban areas only account for a small fraction of the Earth’s land surface, it is important to understand and model the carbon cycle of urban ecosystems as they are a unique anthropogenic ecosystem with intense human influence^3–5^. However, quantifying and assessing the urban carbon cycle faces many challenges^6^. For example, increases in impervious surfaces in urban areas often reduce vegetation coverage, which may affect energy and carbon budgets^5,7–9^. Human activities within urban areas alter the atmospheric environment in both urban and neighboring areas^10–12^. Vegetation within urban areas may have stronger vegetation growth and more carbon uptake^13,14^. Altogether, it is imperative for scientists to better understand the effect of urban expansion on the terrestrial carbon cycle in urban areas.

The gross primary production (GPP) of vegetation is an important variable in the terrestrial carbon cycle. Among the many diagnostic and prognostic biogeochemical models, the Vegetation Photosynthesis Model (VPM) estimates daily GPP (GPP_vpm_) based on satellite images and climate data^15,16^. Over the past decade, VPM has been widely used to estimate the GPP of various ecosystems^17–19^. The resultant GPP_vpm_ estimates have also been compared with GPP estimates from CO_2_ eddy flux sites (GPP_EC_) and other GPP products, and GPP_vpm_ has performed reasonably well at both site and regional scales^20,21^. However, GPP_vpm_ has not yet been evaluated in urban areas because there is no appropriate observation data available for validation.

As one cannot directly measure GPP from space, direct measurements of solar-induced chlorophyll fluorescence (SIF) can serve as a physiological indicator or proxy for photosynthesis and GPP^22,23^, because photosynthetically active radiation (PAR) absorbed by chlorophyll (APAR_chl_) in leaves takes one of three pathways: photosynthesis, chlorophyll fluorescence, and heat^24^. A number of studies have used SIF data to evaluate the phenology of forests, grasslands, and croplands. The results from these studies show that GOME-2 SIF data clearly captures the physiological processes and phenological changes^20,25–27^. Recently, SIF data derived from GOME-2 satellite observations was made available to the public and has a spatial resolution of 0.5° (latitude/longitude) and a monthly temporal resolution^28,29^. The new SIF dataset can be directly compared with GPP estimates, which resolves observational constraints in the analysis of urban GPP^30^. Several studies have compared SIF data with GPP data from tower measurements or individual gridcells^22,25,27,31^. A few other studies have evaluated the relationship between SIF and GPP in different regions^20,23,27,32^. However, until this study, the relationship between SIF and GPP in urban areas has not been evaluated.

One challenge in evaluating the relationship between GPP and SIF in urban area is related to the coarse spatial resolution of the GOME-2 SIF data (0.5° × 0.5°). There are also several different definitions of “urban area”^33–36^, which poses another important challenge^10^. Urban areas can be delineated by administrative boundaries, built-up areas, impervious surface areas^34^, or population size (American Census-Bureau, Urban Area Criteria for the 2010 Census). However, due to the continuous gradients and patch complexity between urban and rural areas, urban areas may encompass rural areas, too. In addition, although finer spatial resolution data can reflect more details, it is hard to distinguish various function-related urban land types, which may be located far away from urban areas^37,38^. Most importantly, the impacts of urban areas on ecosystems extend far beyond the exact urban boundary^3,39^. Accordingly, instead of using urban boundaries (polygon data), some researchers have analyzed grid-based data and found that both urban heat islands and their effects on vegetation phenology in and around urban areas can be successfully identified based on 0.5° × 0.5° gridcells^40^. Using a similar approach, this study describes the urban and surrounding areas as an “urban” gridcell comprising urban, sub-urban, as well as any adjacent rural areas that usually have a high degree of socioeconomic and functional integration with neighboring urban areas.

**Figure 1:**
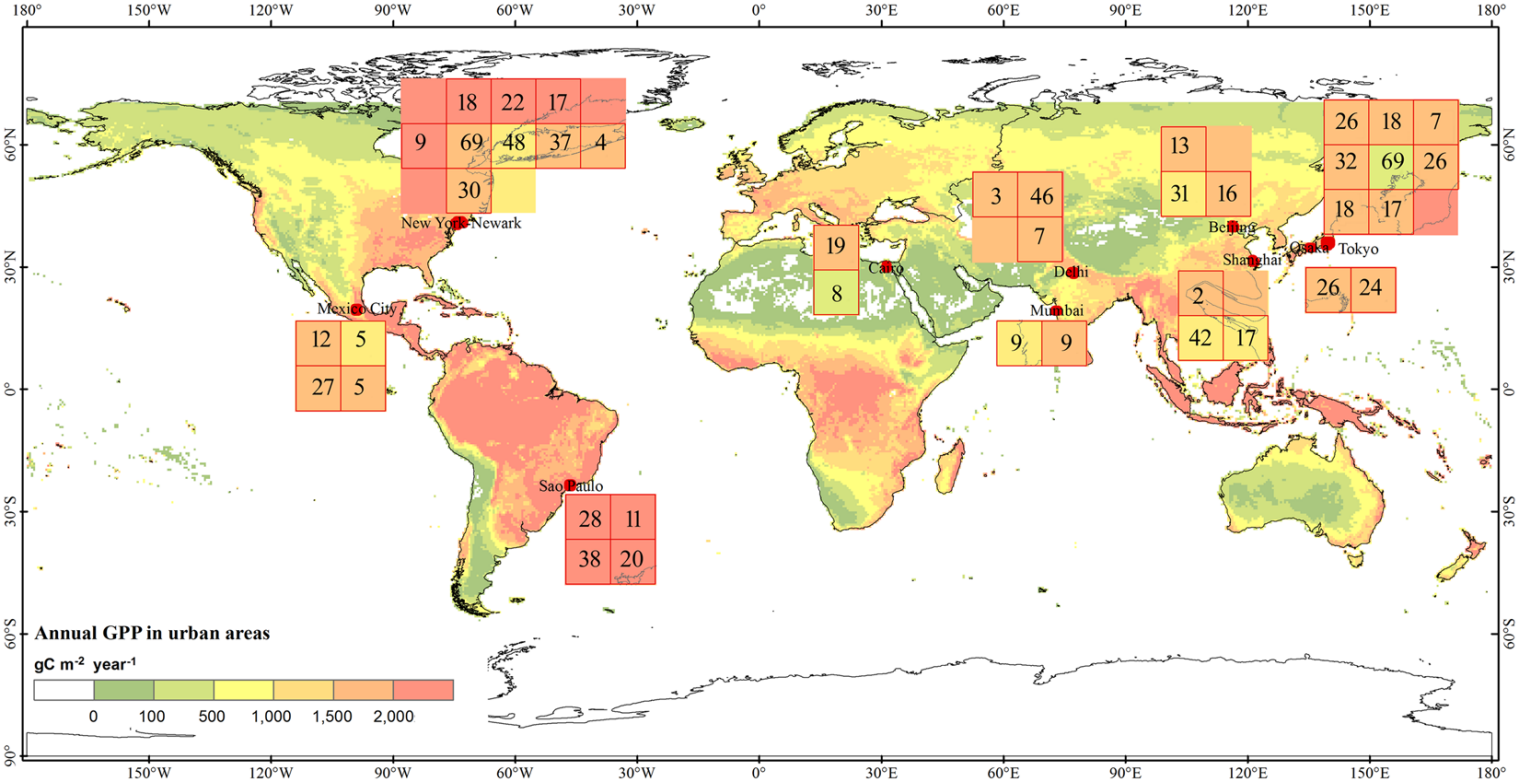
The geographic distributions of the ten most populous megacities in the world: Tokyo (Japan), Osaka (Japan), Beijing (China), Shanghai (China), Delhi (India), Mumbai (India), São Paulo (Brazil), Mexico City (Mexico), Cairo (Egypt), and New York–Newark (USA). These megacities are within two to nine gridcells of 0.5° (latitude/longitude) spatial resolution. These ten cities comprise a total of 40 gridcells. The numbers within individual urban gridcells (red frames) represent the urban area percentage (%) within a gridcell, derived from the land cover map used in the GPP_mod17_ data product^53,55^. The color within the urban gridcells indicate the annual GPP in 2000. Map was generated using ArcGIS 10.1 software (http://www.esri.com/arcgis/about-arcgis).

The objective of this study is twofold: (1) quantify the temporal consistency between seasonal dynamics of GPP data and GOME-2 SIF data at 0.5° spatial resolution in urban areas during 2007–2014, and (2) investigate the interannual variation and trends of annual GPP during 2000–2014 in urban areas. Considering the coarse spatial resolution of GOME-2 SIF data, we selected megacities for our analysis. Megacities are assumed to have relatively large proportions of urban areas within the 0.5° gridcells, which helps ensure that the seasonal dynamics and interannual variation of GPP and GOME-2 SIF data from urban areas are well captured. In this study, we used urban population data from 2015 provided by the latest World Urbanization Prospects (WUP) revision to choose the ten most populous megacities in the world^1^. These megacities are Tokyo (Japan), Delhi (India), Shanghai (China), São Paulo (Brazil), Mumbai (India), Mexico City (Mexico), Beijing (China), Osaka (Japan), Cairo (Egypt), and New York–Newark (USA) (see Fig. 1 and Fig. S1).

## Results

### The seasonal dynamics of GPP and SIF in the ten megacities during 2007–2014

**Figure 2:**
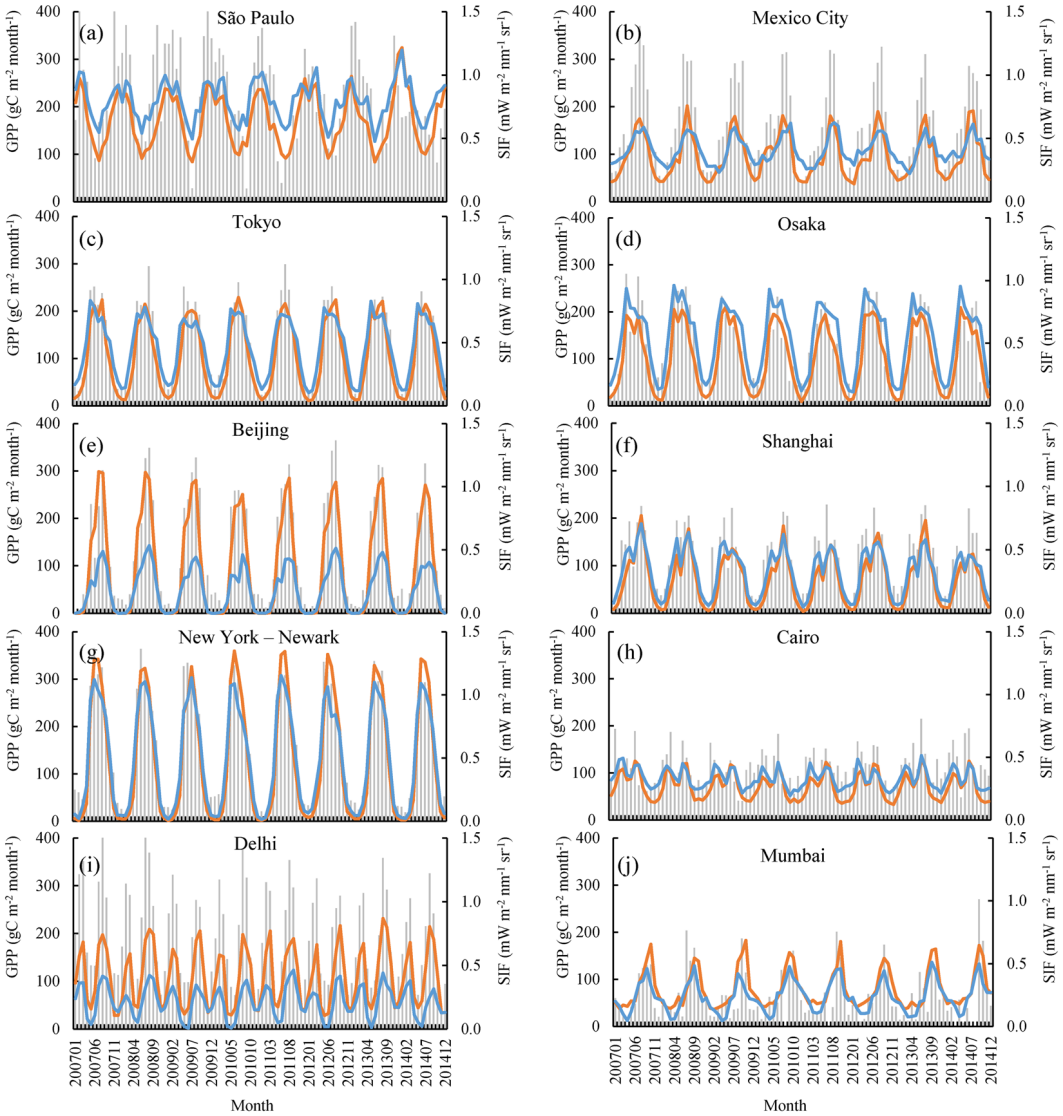
Seasonal dynamics and interannual variation of GOME-2 SIF, GPP_vpm_, and GPP_mod17_ for the ten megacities during 2007–2014. The light gray bar represents GOME-2 SIF; the orange line represents GPP_vpm_; and the blue line represents GPP_mod17_.

Three datasets (GPP_vpm_, GPP_mod17_, and GOME-2 SIF) generated from various data resources and methods are used to examine the seasonal dynamics of urban GPP and SIF. The ten megacities are located in a variety of climate systems: tropical (São Paulo, Mexico, Mumbai), subtropical (Delhi), warm temperate (Shanghai), cold temperate (Beijing, New York–Newark, Tokyo, and Osaka), and arid (Cairo). Figure 2 shows the seasonal dynamics of GOME-2 SIF, GPP_vpm_, and GPP_mod17_ during 2007–2014. SIF data captures distinct seasonality for each of the ten megacities. SIF values are low in the winter (close to 0) or dry seasons, but high in the summer or wet seasons. These seasonal variations clearly illustrate changes in phenology and photosynthesis during the year. The seasonal dynamics of GPP_vpm_ track well with that of GOME-2 SIF data for all ten megacities (Fig. 2 and Fig. S3). Likewise, the seasonal dynamics of GPP_mod17_ also track well with that of GOME-2 SIF data for the ten megacities.

**Figure 3:**
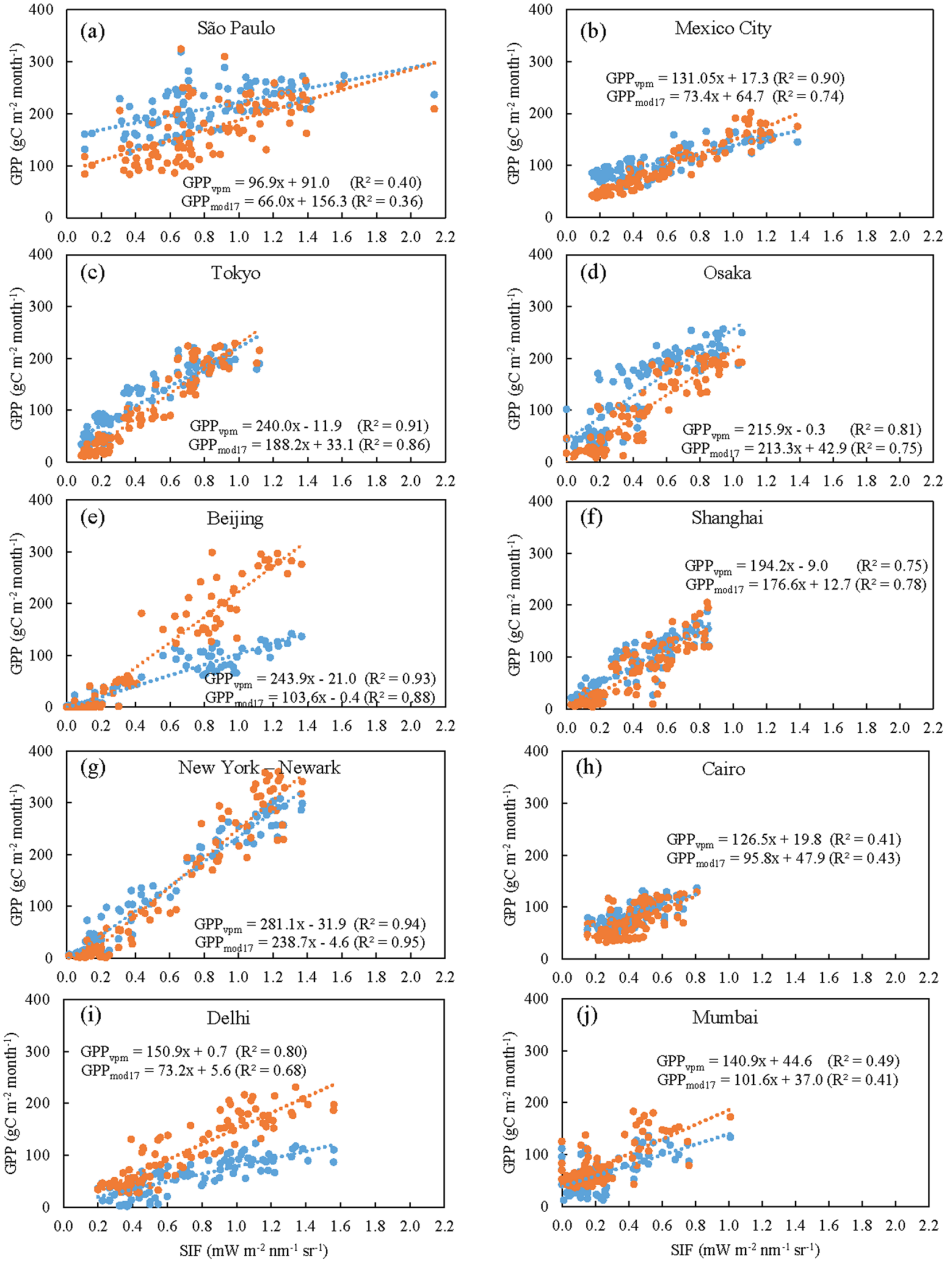
Scatterplots of monthly GPP_vpm_ and GPP_mod17_ versus SIF in the world’s top ten megacities during 2007–2014. Orange dots and fit-lines represent the relationship between SIF and GPP_vpm._ Blue dots and fit-lines represent the relationship between SIF and GPP_mod17_. The results of each analysis are statistically significant (*n = *96, p < 0.001).

The scatterplots of GPP versus SIF during 2007–2014 (Fig. 3) show strong positive correlations between SIF and GPP at the monthly scale. The Pearson’s correlation coefficient between SIF and GPP_vpm_ for each individual megacity ranges from 0.63 in São Paulo to 0.97 in New York–Newark (Table S1). SIF and GPP_vpm_ show strong linear relationships for the ten megacities (Fig. 3 and Fig. S3). Seven megacities have slightly higher coefficients of determination (R^2^) from SIF and GPP_vpm_ analyses than those from SIF and GPP_mod17_ analyses (Fig. 3). For all ten megacities, the slopes of the simple linear regression models from SIF and GPP_vpm_ analyses are larger than those from SIF and GPP_mod17_ analyses. Seven megacities have much smaller absolute intercept values from SIF and GPP_vpm_ analyses than from SIF and GPP_mod17_ analyses. These results indicate that the temporal consistency between SIF and GPP_vpm_ is slightly stronger than that between SIF and GPP_mod17_.

### Interannual variation of GPP in the ten megacities during 2000–2014

**Figure 4:**
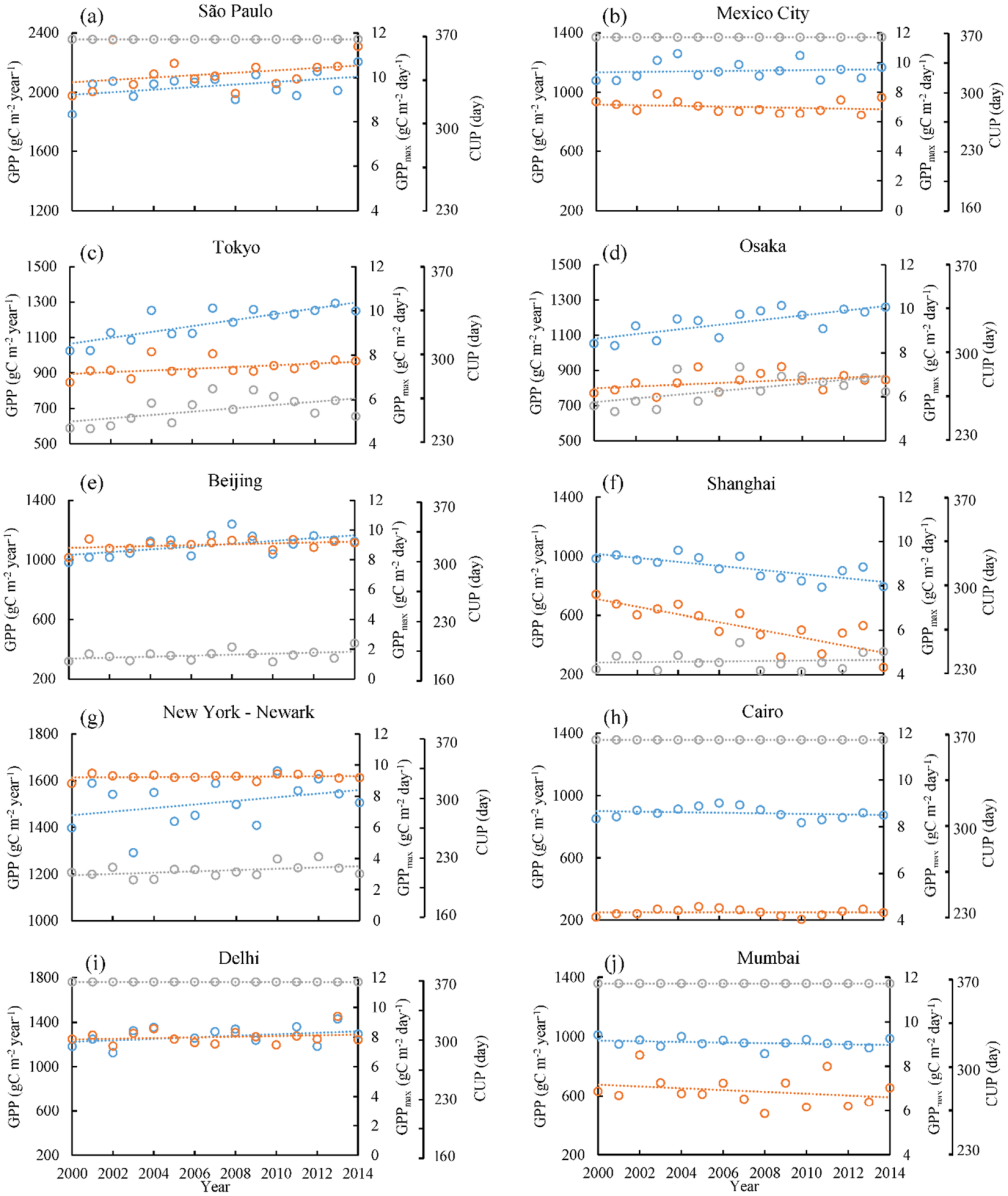
Interannual variations of annual GPP (blue), daily maximum GPP (GPP_max_, orange), and carbon uptake period (CUP, grey) in the world’s top ten megacities from 2000 to 2014. GPP_max_ is the maximum daily GPP in a year; CUP is defined as the number of days with GPP >= 1.0 gC m^−2^ day^−1^ in a year^41,42^; and GPP_max_ is estimated by the Savitzky-Golay fitting method^59^.

**Figure 5:**
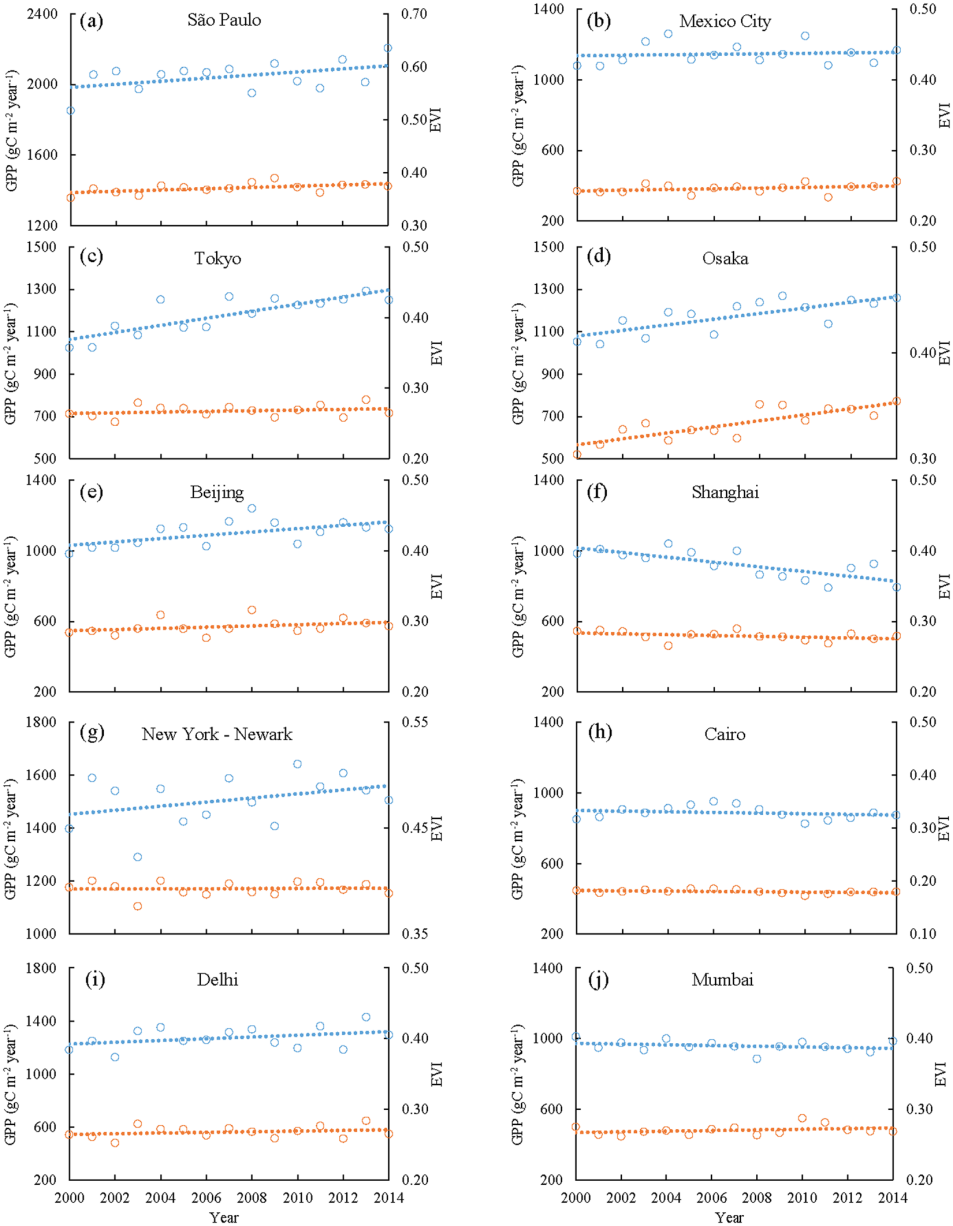
Interannual variations of annual GPP (blue) and Enhanced Vegetation Index (EVI) (orange) in the ten megacities from 2000 to 2014. EVI is the mean EVI within a plant growing period (from April to October or from January to December).

As GPP_vpm_ contains urban GPP estimates from all 500-m pixels within a 0.5° gridcell and GPP_vpm_ has a stronger linear relationship with SIF data, we used GPP_vpm_ data to analyze interannual variations of annual GPP from 2000 to 2014 (Figs 4 and 5). Annual GPP (gC m^−2^ year^−1^) varies substantially among the ten megacities. São Paulo, Brazil, has the highest annual GPP, ranging from ~2,000 to ~2,200 gC m^−2^ year^−1^. New York–Newark, USA, ranks second in annual GPP. Cairo, Egypt, has the lowest annual GPP, approximately ~950 gC m^−2^ year^−1^. The interannual variations in annual GPP differ substantially among the ten megacities. Tokyo and Osaka in Japan have statistically significant linear increases in annual GPP, while Shanghai, China has statistically significant linear decreases in annual GPP. Beijing, China has a strong increase in annual GPP during 2000–2008, but a moderate decrease during 2009–2014, resulting in a moderate increase in annual GPP over the 15 years. The other six megacities do not show increasing or decreasing trends in annual GPP during the 15-year period.

**Figure 6:**
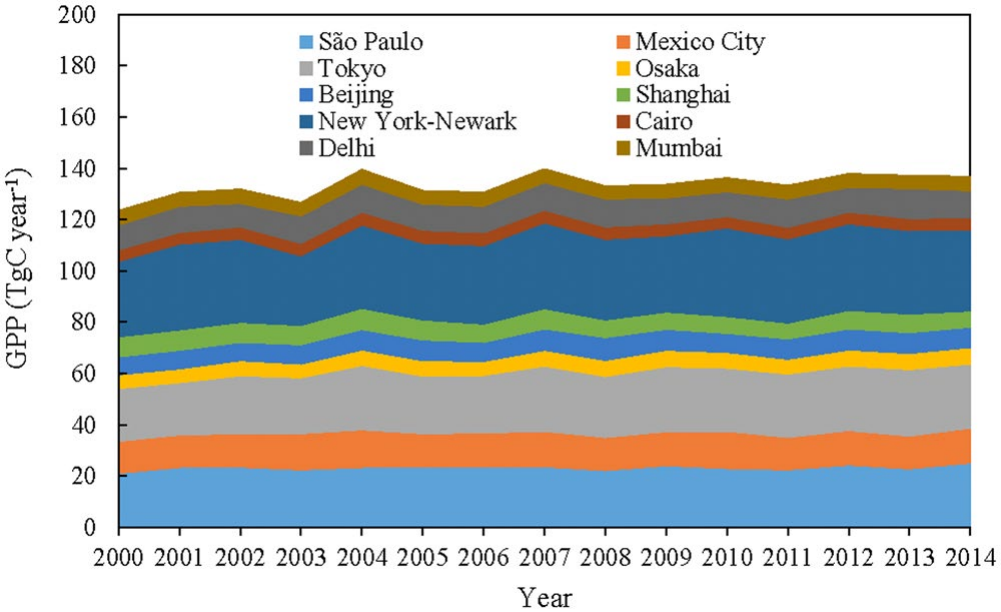
Interannual variation of annual gross primary production (TgC year^−1^) in the ten largest megacities from 2000 to 2014.

We calculated total annual GPP (TgC year^−1^) for each megacity during 2000–2014 as the product of mean annual GPP (gC m^−2^ year^−1^) and the total area of the megacity’s gridcells (Fig. 6). New York–Newark has the largest annual GPP (31.69 TgC year^−1^), followed by Tokyo (23.67 TgC year^−1^), mainly due to their large expanse spanning several gridcells. São Paulo ranks a close third (23.19 TgC year^−1^) due to its highest annual GPP per unit area. Shanghai has experienced the largest loss of annual GPP, from 7.78 in 2000 to 6.28 TgC year^−1^ in 2014. Tokyo has experienced the largest gain in total annual GPP, from 20.53 in 2000 to 25.05 TgC year^−1^in 2014. These ten megacities have a total land area of 103.4 thousand km^2^ (Table S1). The total annual GPP of the ten megacities increased from 123.55 in 2000 to 136.85 TgC year^−1^ in 2014 (Fig. 6 and Table S2), with a mean value of 133.53 TgC year^−1^ over the 15-year period. We also compared the total gridcell areas of the ten megacities with the global total land area (excluding the Antarctic continent and Greenland). The megacities cover around 0.08% of land surface, and their combined GPP comprises around 0.11% of global total GPP (about 120–130 PgC year^−1^). All these results show that urban GPP is important for the study of carbon cycle in urban areas.

### Factors affecting annual GPP within a megacity during 2000–2014

Several studies have suggested that annual GPP is jointly controlled by vegetation phenology and physiology, as measured by maximum daily GPP (GPP_max_) in a year and the carbon uptake period (CUP)^41–43^. We investigate to what degree vegetation phenology (using CUP as a proxy), physiology (using GPP_max_ as a proxy), and greenness (using mean Enhanced Vegetation Index (EVI) of growing season as a proxy) affected the dynamics of annual GPP during 2000–2014 (Figs 4 and 5, and Fig. S4). Each of the three variables show significant correlations with annual GPP (Table S3). For those megacities in tropical and sub-tropical climate zones (São Paulo, Cairo, Mexico City, Mumbai, and New Delhi), where air temperature is above freezing throughout the year and vegetation is mostly evergreen, CUP remained unchanged over the 15-year period. For megacities in temperate and cold-temperate climate zones (Tokyo, Osaka, Shanghai, Beijing, New York–Newark), CUP showed various positive trends during 2000–2014, ranging from 0.15 day year^−1^ in Shanghai to 1.48 day year^−1^ in Osaka. The dynamics of daily maximum GPP (GPP_max_) varied substantially among the 10 megacities with positive trends (São Paulo, Osaka, Tokyo, Beijing, New Delhi), no trend (New York–Newark, Cairo), and negative trends (Shanghai, Mumbai, Mexico City). EVI had a strong increasing linear trend in Osaka, but no other megacity showed clear EVI trends (Figs 4,5 and Table S3).

We further correlated annual GPP with population data (Fig. S5) and nighttime lights data (Figs S2 and S6), which have great potential in mapping urbanization dynamics^44^. The population and nighttime lights data show increasing trends in the ten megacities over time, but the rates of increase vary substantially. The megacities with higher rates of population increase also have higher rates of increase in nighttime lights. GPP, GPP_max_, CUP, and EVI had increasing trends in Tokyo and Osaka, where populations and nighttime lights only increased slightly. In comparison, Shanghai had decreasing trends for GPP_max_ and EVI, but a slightly increasing trend for CUP (0.15 day year^−1^) and large increasing trends in population and nighttime lights (Figs S5, S6; Table S4). Overall, each of the three urban expansion variables (population trend, nighttime lights trend, and urban area percentage) have weaker correlations and less significance with annual GPP than relationships between GPP and GPP_max_, CUP, and EVI.

**Figure 7:**
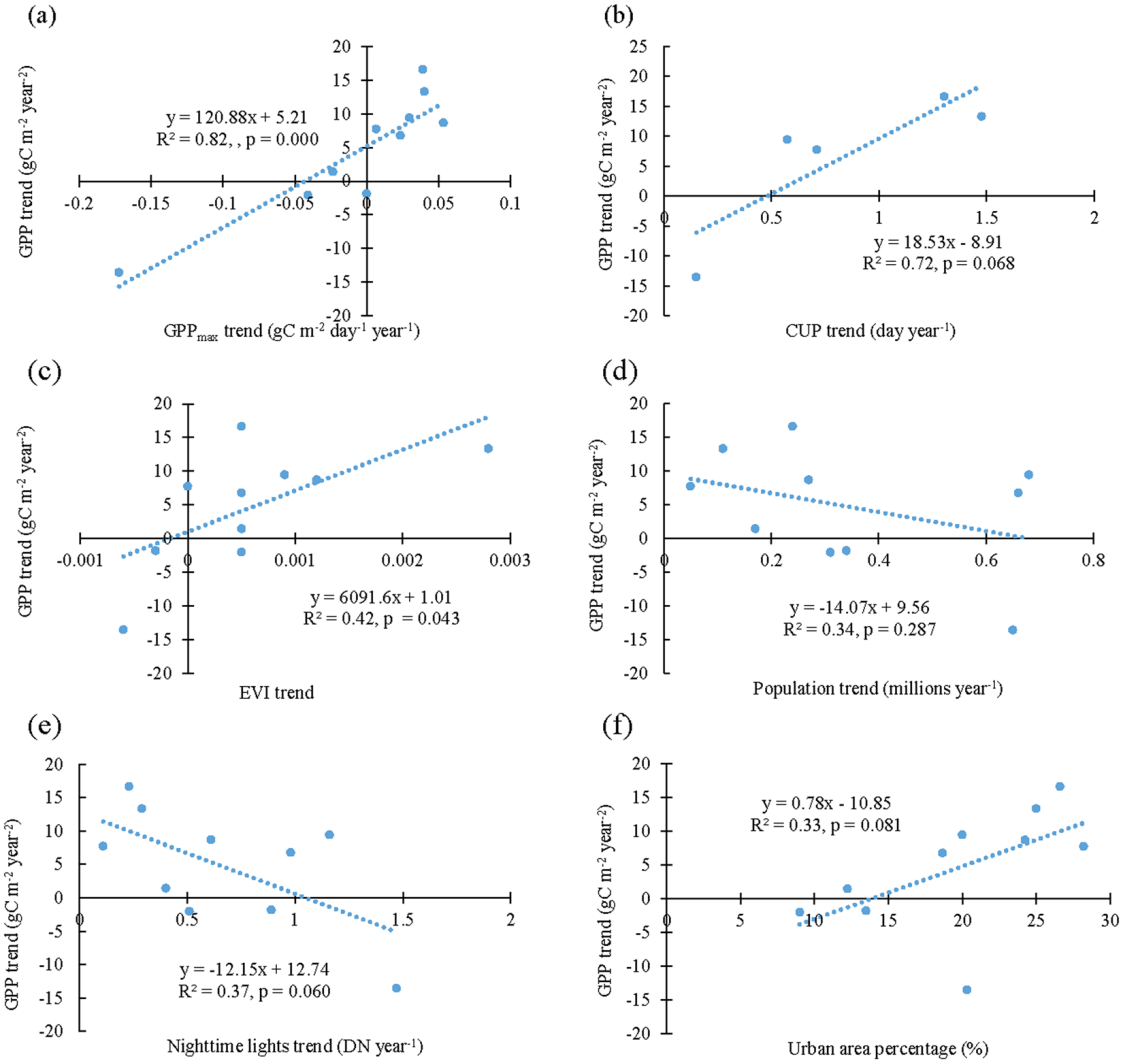
The quantitative relationships between annual GPP trend and GPP_max_, CUP, EVI, population, and nighttime lights trends during 2000–2014 as well as urban area percentage in 2000 (which represented the initial condition for this urban analysis).

In addition, we examined the relationships between the mean annual GPP trend and the trends of GPP_max_, CUP, EVI, population, and nighttime lights in the ten megacities (Fig. 7). There are positive correlations between the annual GPP trends and the GPP_max_, CUP, and EVI trends (Fig. 7a,b,c). Both the population and nighttime lights trends are negatively correlated with the annual GPP trend (Fig. 7d,e). The plot between the annual GPP trend and the urban area percentage in 2000 (Fig. 7f), which represents the initial condition of urbanization in the study, shows that the annual GPP trend during 2000–2014 was not affected by the initial urban area percentage in 2000.

## Discussion

### The seasonal dynamics of SIF and GPP in the ten megacities

Several studies have reported on the relationship between GPP and GOME-2 SIF data for various biomes^22,27,45^. Other studies have also used SIF data to estimate GPP of croplands and forests by developing models between SIF data from GOME-2 and GPP data from eddy flux tower sites^22,31^. GPP_mod17_ and GPP_vpm_ are mainly estimated by light use efficiency (LUE) and APAR. APAR can be obtained by multiplying PAR by the fPAR. The formula can be described as: “*GPP* = *LUE* × *APAR*”. Similarly, we can conceptualize SIF by multiplying APAR by the emitted SIF per photon absorbed (SIF_yield_)^22,31^: “*SIF* = *SIF*_*yield*_ × *APAR*”. GPP and SIF are both proportional to absorbed light (PAR and fPAR), and both estimate the seasonal characteristics of vegetation in the ten megacities. To some extent, this inherent relation may explain why the Pearson correlation coefficient of SIF and GPP seems to increase with latitude (Fig. 3 and Table S1), given the larger seasonality of PAR and fPAR at higher latitudes.

Determining how to evaluate GPP of urban areas is a challenge for all satellite-based models and process-based models, due to a lack of eddy flux tower sites within the urban area. Given the complex and mixed landscapes in an urban area, we decided to use GOME-2 SIF data at 0.5° spatial resolution to carry out the minimum verification of GPP_vpm_. Direct comparisons between SIF and GPP are often constrained by the spatial mismatch between the footprint sizes of CO_2_ eddy flux tower sites (often within a radius of several hundreds of meters) and the 0.5° × 0.5° gridcell^46^. One common approach to address this spatial mismatch issue is to aggregate GPP data from 500-m spatial resolution to 0.5° gridcells to match SIF data. A recent study produced by our research group used this approach and reported strong linear relationships between SIF and GPP_vpm_ for various biomes in North America^20^. In this study, we also aggregated GPP_vpm_ at 500-m spatial resolution into 0.5° gridcells. GOME-2 SIF data showed strong seasonal dynamics of vegetation photosynthesis within megacities (often containing a mixture of various vegetation types) in climatic zones that range from tropical to cold temperate. Our results show good temporal consistency between seasonal SIF and seasonal GPP_vpm_ data in the ten megacities during 2007–2014 (Figs 2, 3 and Fig. S3). Unlike GPP_mod17_, GPP_vpm_ and SIF all consider the vegetation information in urban areas, and they thus show a slightly stronger consistency, especially in summer, when vegetation grows lushly and GPP reach its maximum. Collectively, these studies suggest that GPP_vpm_ data can be used to study the seasonal dynamics of GPP in urban areas.

### Interannual variation and trends of GPP in the ten megacities

Many studies have reported that the clearing of natural and agricultural landscapes for urban expansion resulted in losses of gross and net primary production of vegetation and soil carbon^4,9,11,47,48^. Vegetation intensification within urban areas, often characterized by re-planting (trees, shrubs, and lawn) and intensive management (e.g., irrigation, fertilization), can increase vegetation cover, annual GPP, plant biomass, and soil carbon. One study reports that urbanization increased grassland carbon pools^13^. Another study demonstrated that urbanization resulted in increased vegetation greenness in many cities in China, as measured by trends in EVI^14^. These studies postulated that temperature, light, and water may prolong the vegetation growing season, thereby increasing total annual photosynthesis and carbon uptake by plants in urban areas^13,39,40,49^.

In this study, the diverse trends of annual GPP among the ten megacities during 2000–2014 clearly highlight the complex impacts of urban expansion, vegetation intensification, and urban environment on urban vegetation and ecosystems. In terms of urban expansion’s effects, Figure S1 illustrates human population growth for each megacity since 1950. When using the classic S-shape urbanization curve (initial stage, acceleration stage, terminal stage)^2^, these megacities differ in their positions in the urbanization curve. Three megacities in developed countries (New York–Newark, Tokyo, and Osaka) can be classified as being in the terminal stage of urbanization and have statistically significant increases in annual GPP during 2000–2014, which are likely driven by the increasing trends of GPP_max_, CUP, and EVI in these three megacities. Although CUP increased during 2000–2014, mean annual GPP decreased substantially in Shanghai and moderately in Mumbai and Cairo. Each of these three megacities were experiencing rapid urban expansion. These results suggest that the increasing growing season length (CUP) is insufficient to offset the negative impact of urban expansion on annual GPP, which is consistent with a recent study focused on natural ecosystems^50^. The initial condition of urban area percentage in 2000 also affects the trends of population and nighttime lights during 2000–2014 (Fig. S7). Overall, the impact of urban expansion on GPP might be partly offset by vegetation intensification and the urban environment.

### Challenges and perspectives

Urban ecosystems, where natural and human processes are interwoven, are perhaps the most complex socio-ecological systems. Our understanding of urban ecosystems and their role in the carbon cycle remains poor^3,10^. Urban landscapes usually comprise both vegetation and artificial impervious surfaces, which have different or even opposing effects on overall urban vegetation productivity. The definition of urban areas remains uncertain because of the complexity of judging the urban spatial extent^38^. Furthermore, urbanization can impact vegetation growth and the carbon cycle beyond the physical boundary of an urban area. It has been reported that rural vegetation outside of urban areas can be influenced by changes in climate due to urbanization^40^. To improve our understanding of urban ecology and the carbon cycle, it is essential to develop multi-scale integrated methods to observe urban ecosystems using satellite-based and process-based models. Our study provides a large-scale analysis method to quantify GPP, one of the key components in the urban carbon cycle, and shows that the GPP within urban gridcells can be estimated by satellite-based VPM. In this study, through analyses of monthly and annual GPP_vpm_, GPP_mod17_, and SIF data during 2007–2014 (0.5° gridcell), we illustrated that both GPP_vpm_ and GPP_mod17_ correlate significantly with GOME-2 SIF data in the world’s ten most populous megacities. GPP_vpm_ and SIF had slightly stronger consistency than did GPP_mod17_ and SIF, partly because both GPP_vpm_ and SIF data contained all the areas within a gridcell. This difference is why GPP_mod17_ was less than GPP_vpm_ in most megacities, especially Beijing and Delhi. The difference may also be due to the low LUE values in the PSN algorithms for the nearby land cover types for Beijing and Delhi. The comparative results showed that GPP_vpm_ can be used to analyze the seasonal dynamics and interannual variation of GPP in urban areas. Moreover, a verification and comparison on VPM and GPP_vpm_ among different land cover types have been done in Beijing (Fig. S8). The results show both VPM and GPP_vpm_ are credible based on our general judgment. The results also confirm the positive effects of urban vegetation on the increased GPP in urban gridcells. However, we still need observation data for urban areas to explore the uncertainty of GPP_vpm_, though very little urban area data is currently available. Nevertheless, our study demonstrates that the use of urban gridcells is a feasible method to explore the impacts of urbanization on vegetation within urban and surrounding areas.

The diverse interannual dynamics of annual GPP among the ten megacities, which represent a small fraction of urban areas in the world, clearly calls for more comprehensive studies across urban areas of various sizes. The results of this study also demonstrate the potential of satellite-based LUE models and SIF in the study of urban ecosystem physiology and the carbon cycle in urban areas. As new SIF data become available from the OCO-2 satellite, the integration of satellite-based LUE models and OCO-2 SIF will likely provide an improved capacity to measure, monitor and model urban ecosystem physiology, which is vital for us to understand terrestrial carbon cycles at local, regional and global scales.

## Materials and Methods

### Urban area and population dataset

The Global Urban Area dataset (GUAD) was developed by Dr. Seto’s Lab at Yale University (http://urban.yale.edu/data)^51^. The urban area dataset at 5 km × 5 km resolution was spatially aggregated to 0.5° × 0.5° to match the spatial resolution of SIF data from GOME-2. The spatial extent of these ten megacities covered a total of 40 gridcells (Fig. 1). The urban population data in 2015 provided by the latest World Urbanization Prospects (WUP) revision (https://esa.un.org/unpd/wup/) was used to select the ten most populous megacities^1^. Population data for 2000, 2005, 2010, and 2015 were also used to analyze the annual change of urbanization.

### SIF dataset from GOME-2

SIF data from Global Ozone Monitoring Instrument 2 (GOME-2) over the period of 2007 to 2014 were used in this study^25^. The SIF signal is retrieved using a principle component analysis algorithm at wavelengths around 740 nm with a nominal nadir footprint of 40 km × 80 km. We used the latest version (V26) of the GOME-2 SIF product during 2007–2014 with a monthly temporal resolution and 0.5° spatial resolution to assess the seasonal dynamics of vegetation physiology (http://avdc.gsfc.nasa.gov/)^25,28,29^. SIF is directly related to the electron transport process of photosynthesis and therefore is a good indicator of vegetation photosynthetic activity^22,23,27,45^.

### Gross primary production dataset from the Vegetation Photosynthesis Model (VPM)

In this study, GPP data from 2000 to 2014 are predicted using a data driven approach with VPM, surface reflectance data, land surface temperature from Moderate Resolution Imaging Spectroradiometer images (MODIS), and NCEP-Reanalysis II (National Center for Environmental Prediction) climate data^20,52^. With a non-linear spatial interpolation method^53^, the NCEP- Reanalysis radiation and temperature dataset are downscaled from its spatial resolution of around 1.875 ° × 2 ° to 500-m to match with the MODIS data. The VPM uses the fraction of PAR absorbed by chlorophyll to estimate light absorption for photosynthesis. It also uses a satellite-retrieved land surface water index (LSWI) to assess water limitation on photosynthesis^15,16,20^. The GPP_vpm_ product used in this study has a spatial resolution of 500 m and a temporal resolution of 8 days. In order to be consistent with the spatiotemporal resolutions of SIF data, the GPP_vpm_ data is spatially aggregated to 0.5 × 0.5° gridcells and temporally aggregated to monthly and annual time scales. The aggregated GPPvpm data are then used for statistical analyses of GPP and SIF, following similar procedures reported in previous work^20^.

### Gross primary production dataset from the MODIS PSN model (MOD17A2)

As part of the MODIS Land Science data products, the photosynthesis model (PSN) is used to generate a global GPP data product (MOD17A2, GPP_mod17_), which has a temporal resolution of 8 days and a spatial resolution of 1 km^53,54^. In this study, the monthly GPP_mod17_ product with a spatial resolution of 0.05° during 2000–2014 was downloaded from the Numerical Terradynamic Simulation Group (NTSG) website (http://www.ntsg.umt.edu/project/mod17) and then aggregated into a 0.5° grid. Note that the PSN model does not simulate GPP of urban pixels at a 500 m resolution^55^. As the GPP_mod17_ data product did not calculate GPP for urban pixels^54,55^, the NA values of urban areas in GPP_mod17_ were treated as zero based on the hypothesis of no vegetation but impervious surfaces in urban areas. The urban mask in the GPP_mod17_ dataset at 0.05^o^ spatial resolution was used to calculate the percentage of urban area within the 0.5° gridcells (Table S1).

### Enhanced Vegetation Index (EVI) dataset from MODIS/Terra (MOD13C2)

The Monthly Enhanced Vegetation Index (EVI) data from 2000 to 2014 provided by the MODIS/Terra Vegetation Indices dataset (MOD13C2, CMG V006) with a spatial resolution of 0.05° was used to analyze vegetation greenness (ftp://ladsftp.nascom.nasa.gov/allData/6/MOD13C2/)^56,57^. To be consistent with the spatial and temporal resolutions of GOME-2 SIF and GPP data, EVI data were spatially aggregated to 0.5 × 0.5° gridcells and temporally aggregated to monthly and annual time scales.

### Nighttime Lights (NTL) dataset from DMSP-OLS

The Nighttime Lights annual composites dataset (V4) from the DMSP-OLS Nighttime Lights data with spatial resolution of 30 arc-seconds from 2000 to 2013, provided by National Centers for Environmental Information (NCEI, formerly NGDC), can be downloaded at https://www.ngdc.noaa.gov/eog/download.html. The dataset was used as an indicator of urban expansion and intensification^58^. The NTL dataset was aggregated into 0.5^o^ resolution.

## Methods

In this study, only terrestrial GPP was analyzed, and other land cover types, such as ocean, water, etc., have been removed in the original 500 m GPP product by setting values in non-terrestrial and water surfaces to null. Nighttime Lights data, EVI data, and MODIS GPP were handled with the same method. Pearson correlation and linear regression analyses of CUP, GPP_max_, EVI, Nighttime Lights, population, and urban area percentage in 2000 were conducted to evaluate their relative roles in annual GPP trends during 2000–2014. CUP and GPP_max_ are two key factors for GPP. For the analyses of factors driving annual GPP variation, we used the number of days with GPP >  = 1.0 gC m^−2^ day^−1^ in a year to define the carbon uptake period CUP^41,42^ and used the Savitzky-Golay fitting method to obtain the daily maximum GPP in a year (GPP_max_)^59^. It should be noted that CUP is related but not equal to the growth period of vegetation because the time of budburst may not indicate the beginning of the CUP, which usually starts when the leaves are large enough to photosynthesize at a rate higher than respiration rate. Given that the coarse 0.5° gridcells contain mixed ecosystems and limit the capacity for satellite sensors to detect vegetation production, and that we used only GPP instead of net ecosystem productivity (NEP) to quantify CUP, we used a fixed threshold above 1.0 gC m^−2^ day^−1^ to define the CUP based on previous studies^42,43,50^.

## Electronic supplementary material

Supplementary Material
