# Supplementary material for: Temporal consistency between gross primary production and solar-induced chlorophyll fluorescence in the ten most populous megacity areas over years
Source: Sci Rep. 2017 Nov 2;7:14963. doi: 10.1038/s41598-017-13783-5 (PMC5668374; doi:10.1038/s41598-017-13783-5)
Supplement: Supplementary file 1 — Supplementary Material [file 41598_2017_13783_MOESM1_ESM.pdf]

Temporal consistency between gross primary production and solar-induced chlorophyll fluorescence in the ten most populous megacity areas over years

Yaoping Cui[1,2], Xiangming Xiao[2,3,*], Yao Zhang[2], Jinwei Dong[4], Yuanwei Qin[2], Russell B. Doughty[2], Geli Zhang[2], Jie Wang[2], Xiaocui Wu[2], Yaochen Qin[1], Shenghui Zhou[1], Joanna Joiner[5], Berrien Moore III[6]

[1]Collaborative Innovation Center for the "Three Modernization" Harmonious Development of Central Plains Economic Region, Laboratory of Geospatial Technology for the Middle and Lower Yellow River Regions, Henan University, Kaifeng, Henan 475004, China

[2]Department of Microbiology and Plant Biology, Center for Spatial Analysis, University of Oklahoma, Norman, Oklahoma 73072, USA

[3]Ministry of Education Key Laboratory of Biodiversity Science and Ecological Engineering, Institute of Biodiversity Science, Fudan University, Shanghai 222200, China

[4]Institute of Geographic Sciences and Natural Resources Research, Chinese Academy of Sciences, Beijing 100101, China

[5]NASA Goddard Space Flight Center, Greenbelt, MD 20771, USA

[6]College of Atmospheric and Geographic Sciences, University of Oklahoma, Norman, Oklahoma 73072, USA

Correspondence to: Xiangming Xiao (xiangming.xiao@ou.edu)

Supplementary information

A minimal verification of VPM and the impact of buildup areas on GPP within urban gridcells.

The spectral mixture in urban areas suggests that if indices at impervious surface and vegetation surface have been verified individually, their combination should also work. To check the rationality of using VPM to estimate urban GPP, we used the 8-day MODIS data with 500 m resolution to show the seasonal dynamics of key process parameters (vegetation indices and LSWI) for a few MODIS pixels (relatively pure impervious surface, mixed surface, and relatively pure vegetation), so as to illustrate the differences among these land cover types. Here, eight points of Beijing were selected to show the seasonal dynamics of EVI and LSWI, which can directly reflect the fAPAR and light use efficiency (LUE). The results showed that the value of impervious surface was almost minimal among common land cover types during an any year (the year 2010 was showed in Fig. S8), conforming to our general understanding about the EVI and LSWI of impervious surface, mixed surface, and vegetation surface (Fig. S8).

In addition, we took Beijing as an example to check the causes of GPP changes from 2000 to 2014 and distinguish the contribution of buildup areas or their surrounding areas within an urban gridcell on the GPP changes. Figure S8 shows a large difference in mean GPP between buildup areas and their surrounding areas, but the increasing trend of GPP in buildup areas was larger than that in surrounding areas (13.83 > 13.71). It implies that buildup areas can contribute no less than their surrounding areas to the GPP increase within the whole urban gridcells of Beijing. Moreover, the maximum GPP (GPP_{max}) may, to a certain extent, highlight vegetation information under conditions of the best human and natural influences on urban and its surrounding areas. Figure S8 also clearly illustrates that the increasing trend of maximum GPP in buildup areas was much larger than that in surrounding areas (73.35 > 7.96), indicating huge impacts of human management and urban environment on urban vegetation. It also confirmed the energetic effects of buildup areas on the increased GPP in urban gridcells.

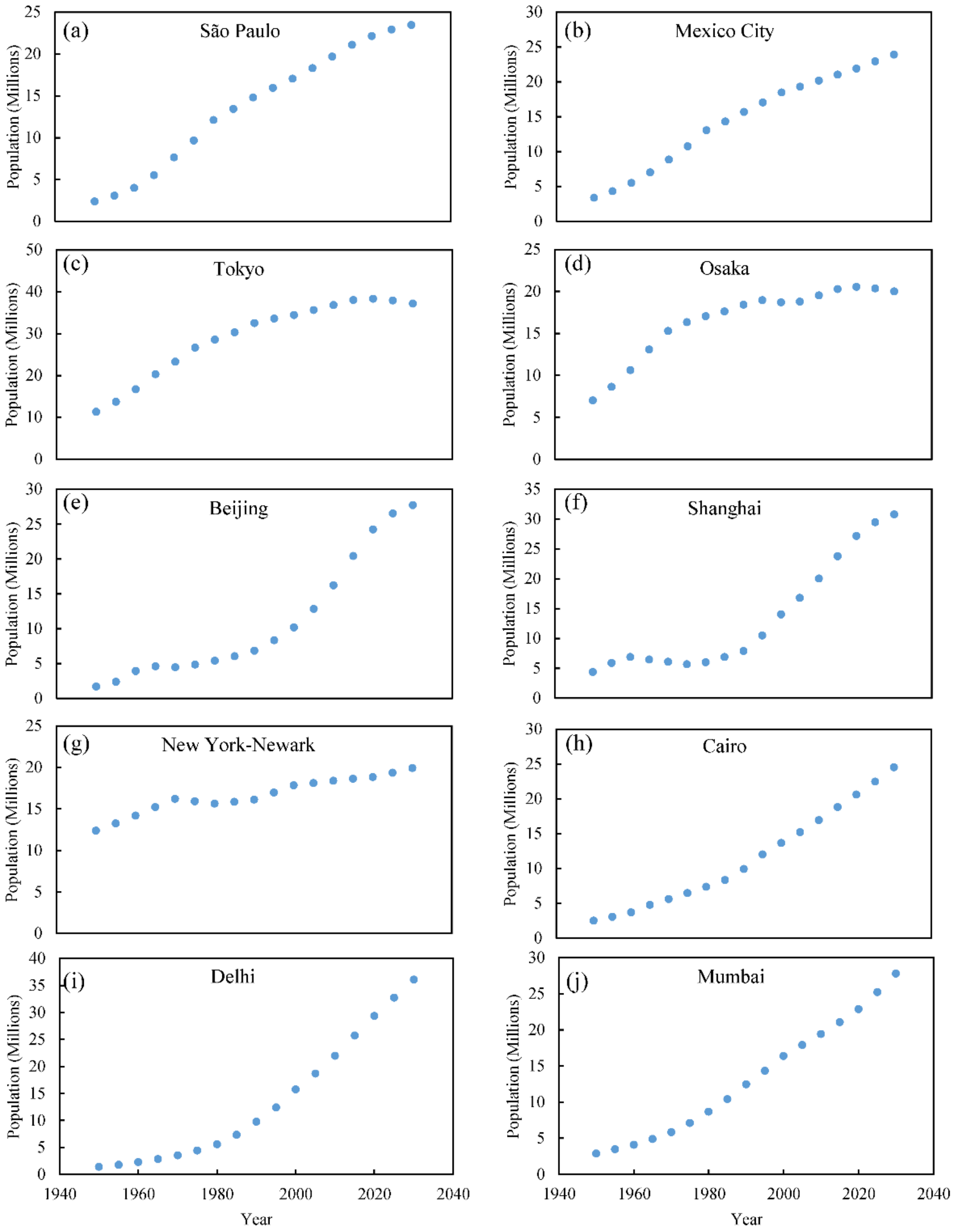

Figure S1. Population variation and urbanization levels in the ten megacities. The population data is from World Urbanization Prospects: The 2014 Revision. All population data can be obtained from https://esa.un.org/unpd/wup/CD-ROM/.

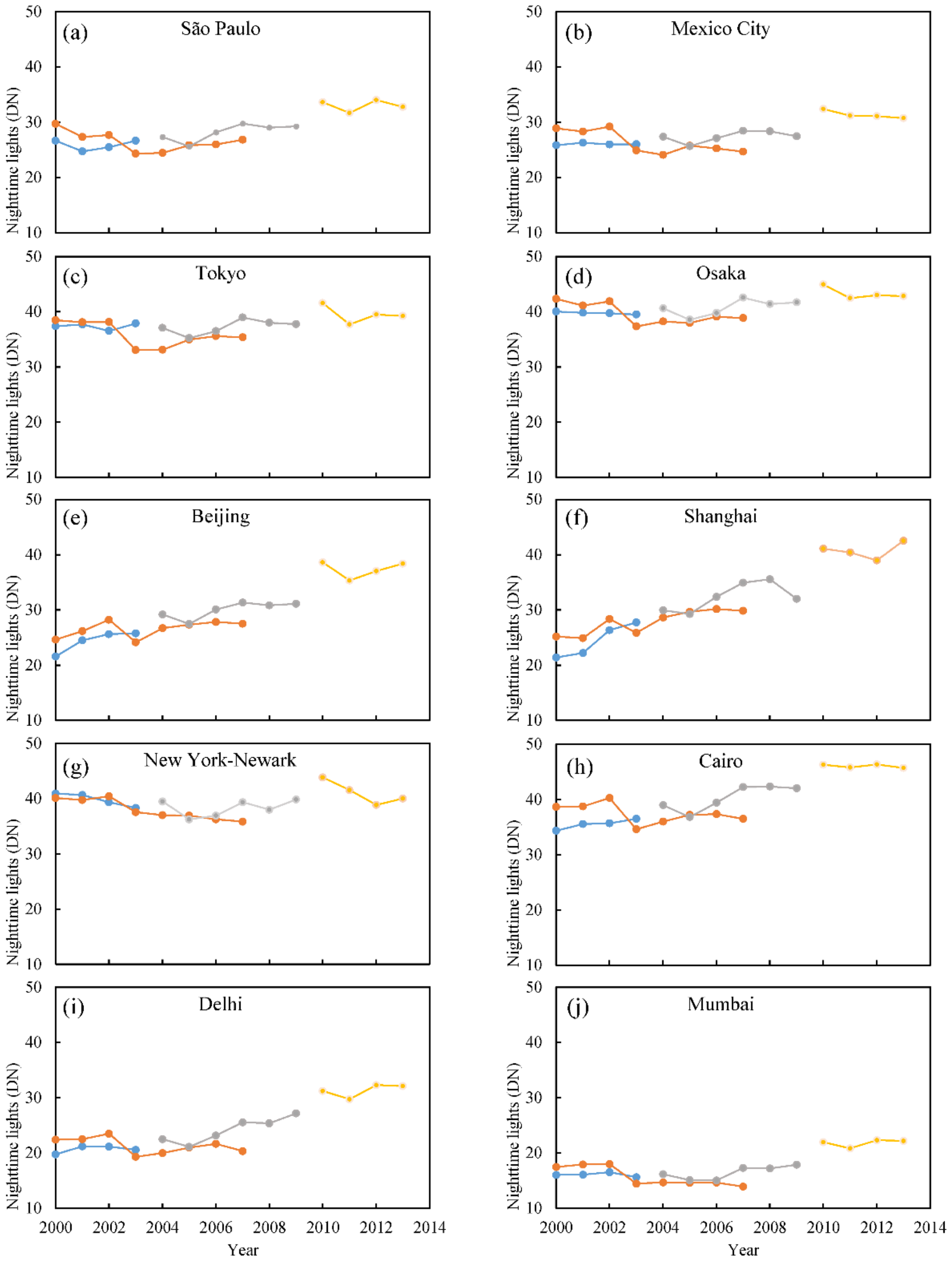

Figure S2. Annual nighttime lights from 2000 to 2013. The annual data come from different satellite products that overlap during the study period. Blue, orange, gray, and yellow dots and lines represent the data resources from F14, F15, F16, and F18, respectively.

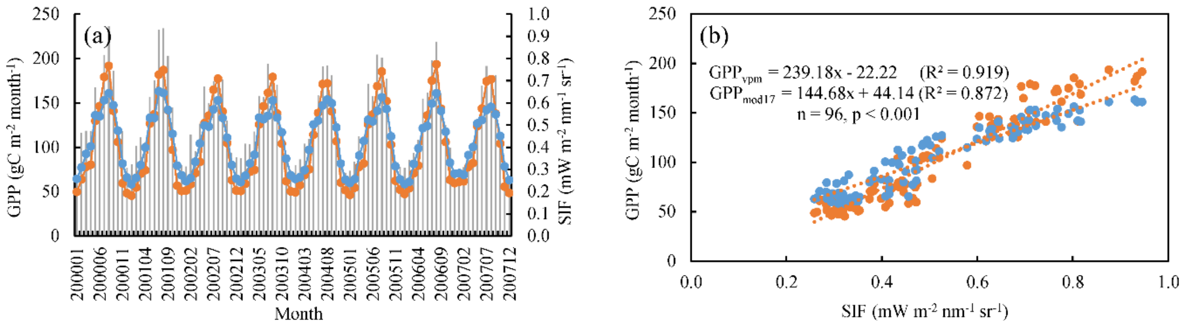

Figure S3. Seasonal dynamics and scatterplot of SIF and GPP from 2000 to 2014. All of the monthly GPP and SIF data for all 40 urban gridcells are averaged. The orange and blue dots and fitting lines represent monthly GPP_{vpm} and GPP_{mod17}, respectively, and the gray columns represent SIF. (a) the seasonal dynamics of mean monthly SIF and GPP (GPP_{vpm} and GPP_{mod17}) in the 10 most populous megacities; (b) monthly mean SIF and monthly sum GPP (GPPvpm and GPPmod17) in the 10 most populous megacities. In Fig. 1a, the x-axis is from January of 2007 to December of 2014. In Fig. 1b, the trend lines of GPP_{vpm} and GPP_{mod17} intersect at approximately 115 gC m^{-2} $month^{-1}$ (or 0.6 mW m^{-2} nm^{-1} sr^{-1}).

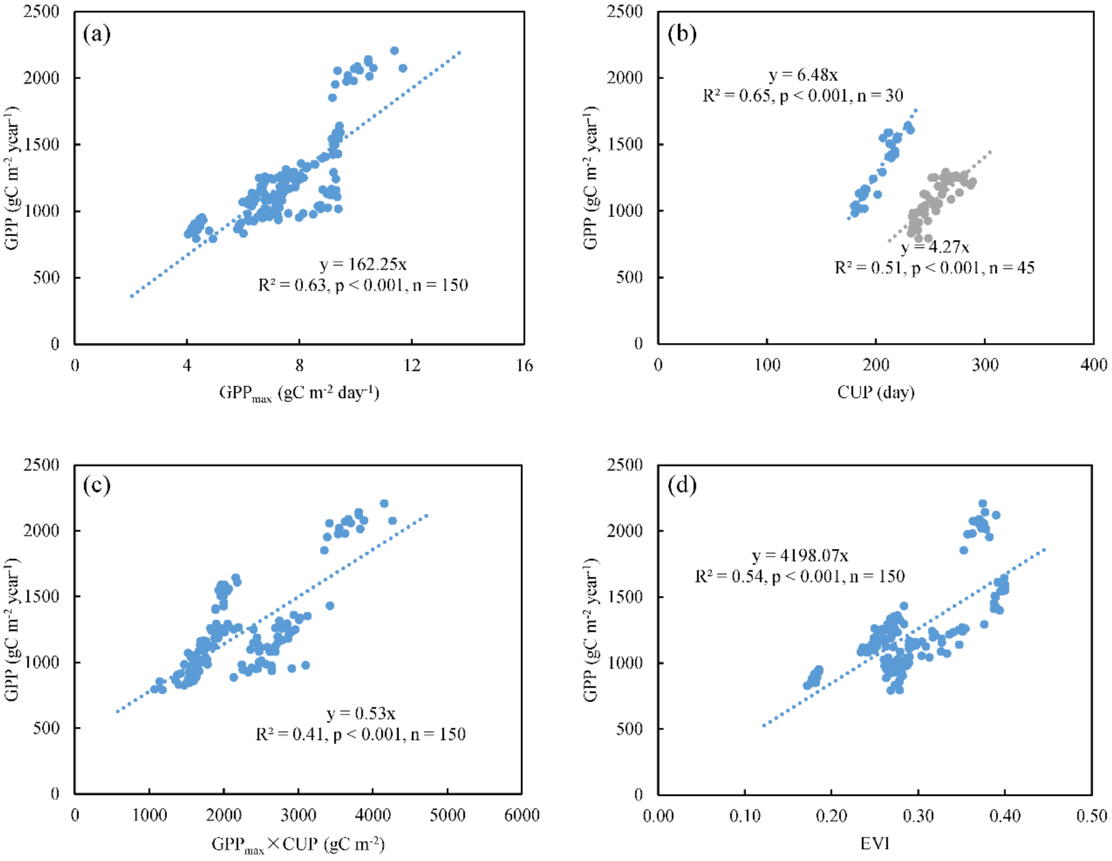

Figure S4. The relationship between annual GPP and GPP_{max}, CUP, GPP_{max}×CUP, and EVI. Unlike other variables, CUP (Fig. S4b) can be divided into two groups according to its geographic location: one is the blue dots, which represent the CUP in New York-Newark and Beijing (cold temperate); the other, gray dots and line are the CUP in Tokyo, Osaka, and Shanghai (cold and warm temperate). In the 5 other megacities, CUP is equal to an entire year because they are located in tropical, subtropical, and arid climates. However, the values of GPP_{max} × CUP in Fig. S4c do not show a similar grouping characteristic.

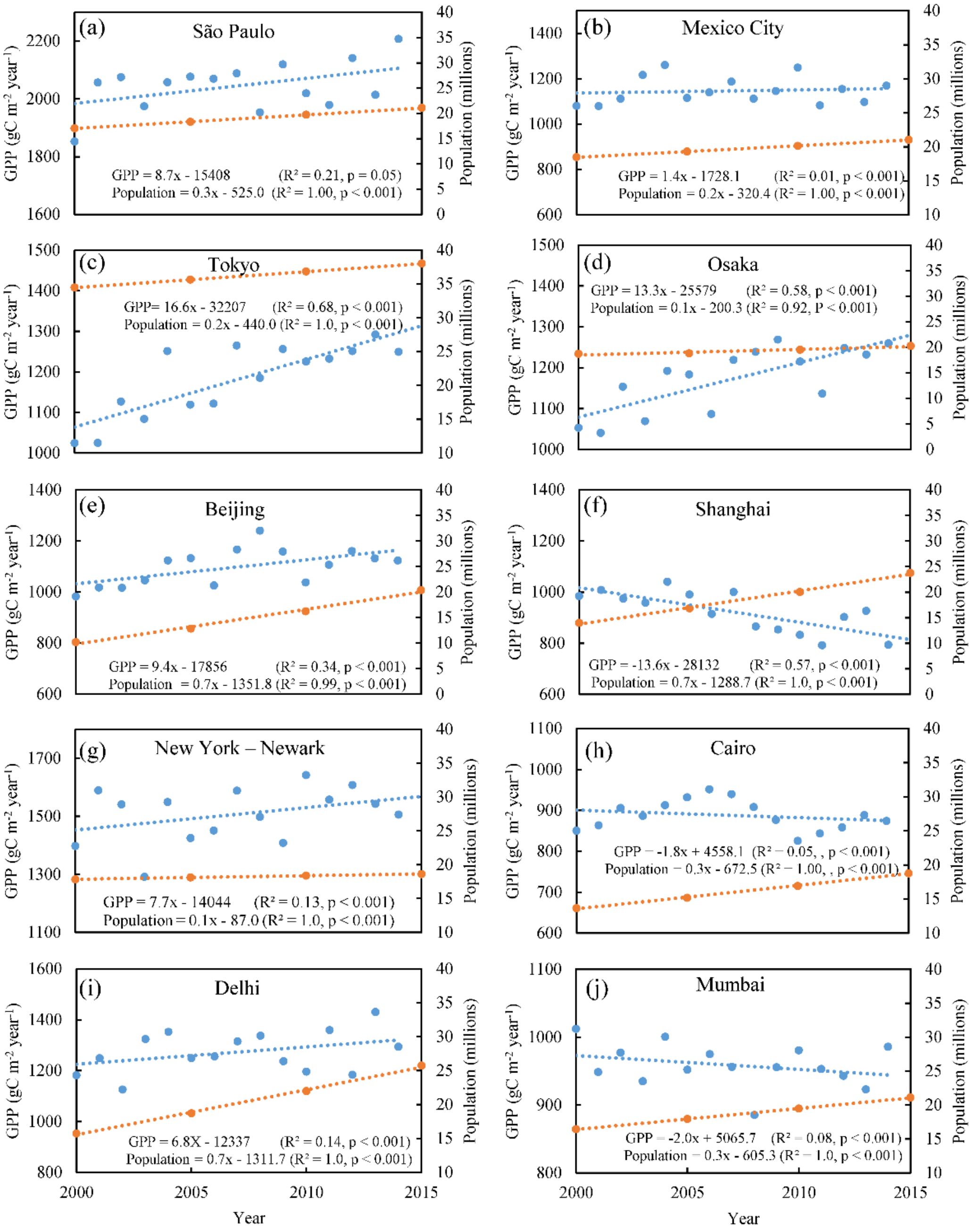

Figure S5. Interannual variations of GPP and population in the ten megacities. Blue dots and fit-lines represent GPP (2000–2014) and orange dots and fit-lines represent population (2000, 2005, 2010, and 2015).

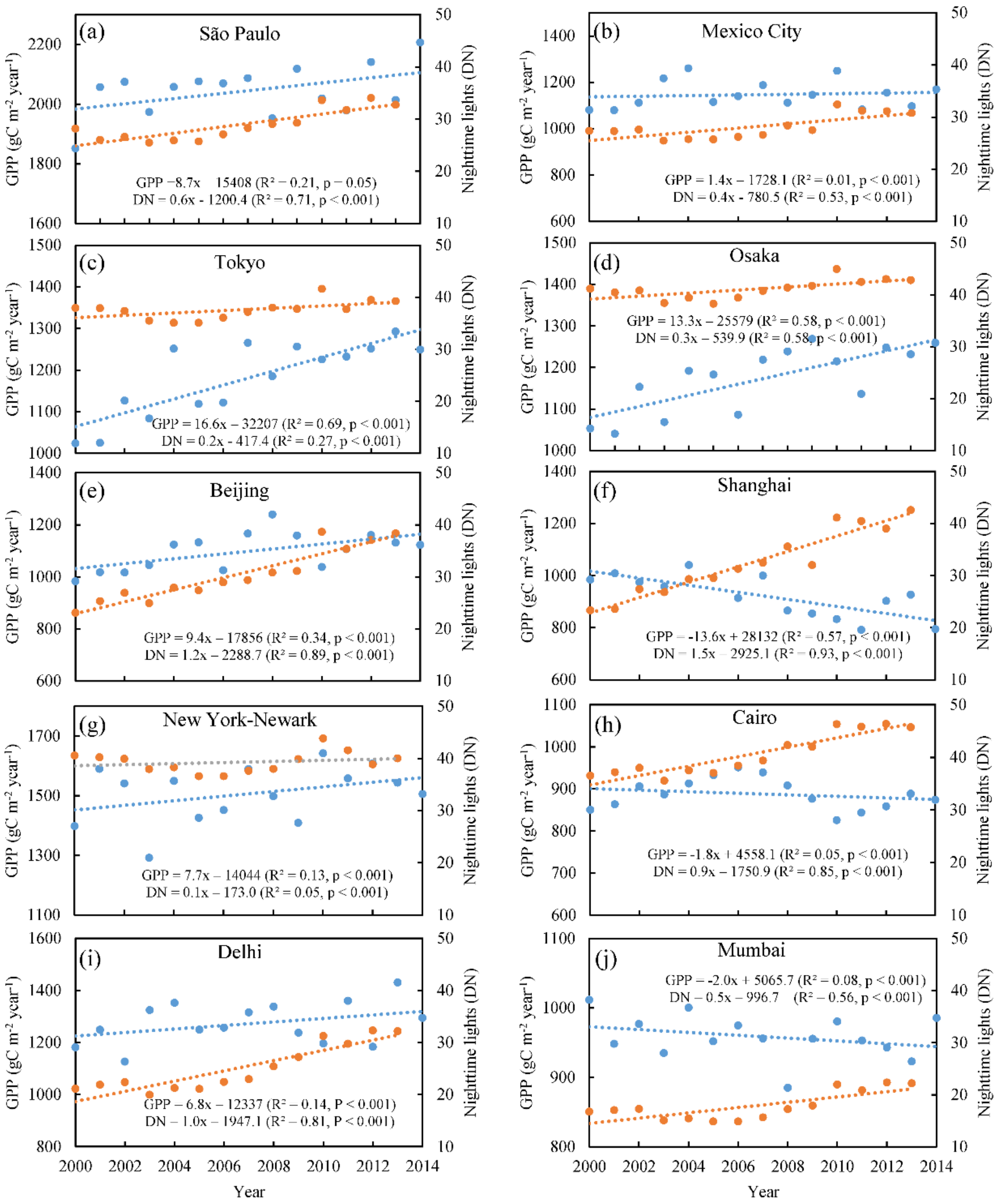

Figure S6. Interannual variations of GPP and Nighttime lights DN in the ten megacities. Blue dots and fit-lines represent GPP (2000–2014) and orange dots and fit-lines represent Nighttime lights DN (2000–2013). The overlapping Nighttime lights data in corresponding years were averaged.

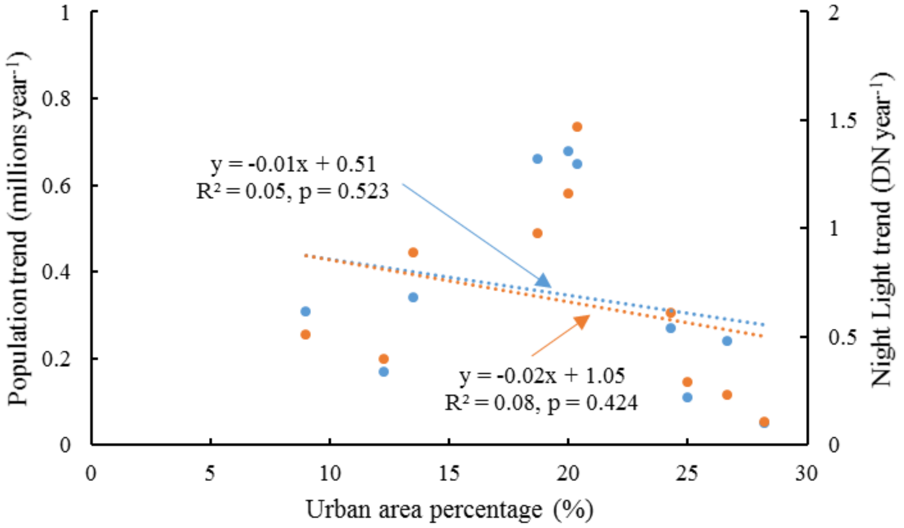

Figure S7. The quantitative relationships between urban area percentage and population trend, and nighttime lights DN trend in the ten megacities. The orange dots and fit-line represent population trend; the blue dots and fit-line are Nighttime lights DN trend (2000–2014).

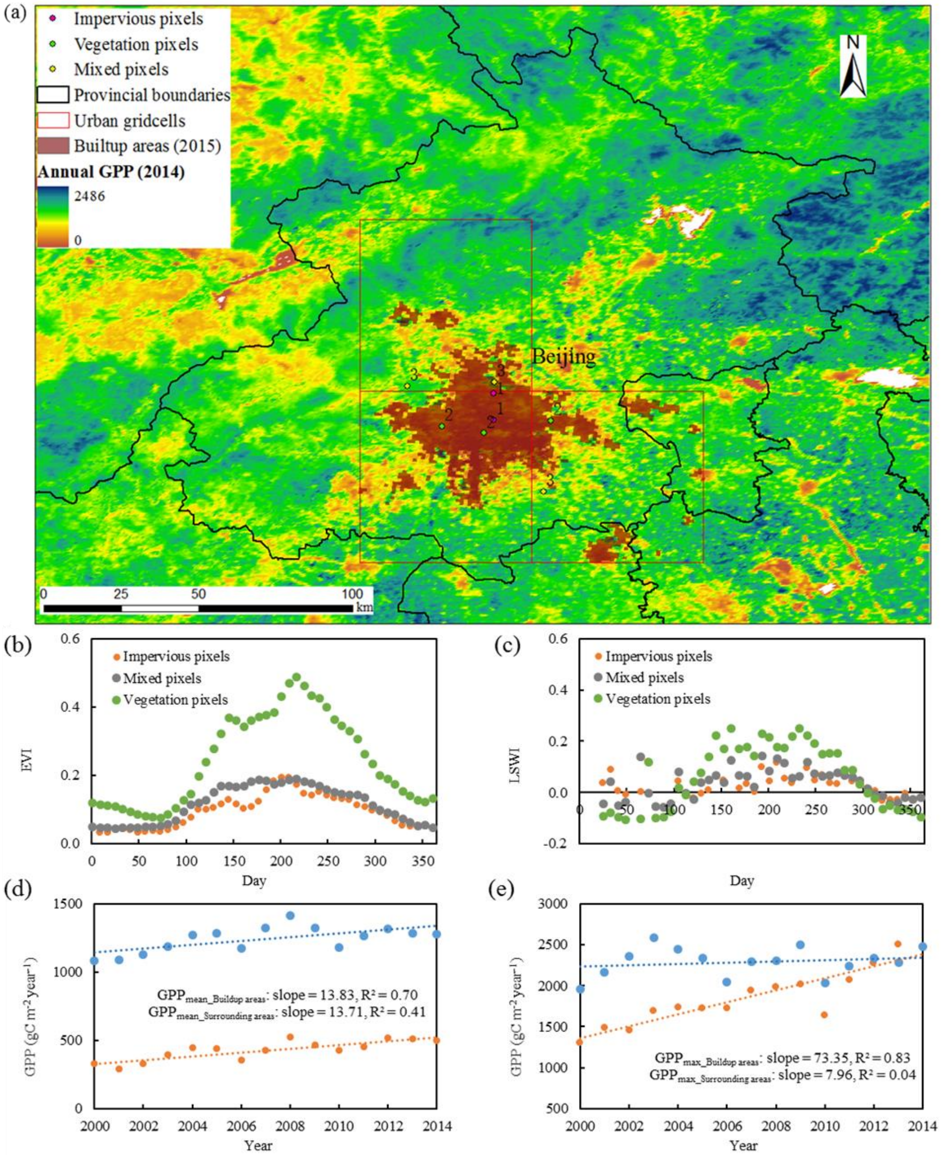

Figure S8. The comparison of buildup areas and their surrounding areas within the urban gridcells. (a) Spatial distribution of eight points and buildup areas in Beijing; (b and c) Seasonal dynamics of process parameters (EVI and LSWI) among common land cover types in urban gridcell in 2010; (d and e) Interannual variations of the mean or maximum GPP in buildup areas and their surrounding areas from 2000 to 2014. Orange dots and fit-lines represent the mean or maximum GPP in buildup areas; blue dots and fit-lines represent the mean or maximum GPP in surrounding areas. Map (a) was generated using ArcGIS 10.1 software (http://www.esri.com/arcgis/about-arcgis)

Table S1. A summary of population, total land area, urban area percentage, and correlation coefficients between SIF and GPP (GPP_{vpm} and GPP_{mod17}) during 2007 – 2014 in the world's ten most populous megacities.

Megacity name	Population (millions)	Number of 0.5° gridcells	Total land area (0.5° gridcell, thousand km^2)	Urban area percentage (%)	Correlation coefficient	
					SIF and GPP_{vpm}	SIF and GPP_{mod17}
São Paulo	21.1	4	11.34	24.25	0.63	0.60
Mexico City	21.0	4	11.66	12.25	0.95	0.86
Tokyo	38.0	8	20.05	26.63	0.95	0.93
Osaka	20.2	2	5.08	25.00	0.90	0.86
Beijing	20.4	3	7.11	20.00	0.96	0.94
Shanghai	23.7	3	7.91	20.33	0.87	0.88
New York–Newark	18.6	9	21.04	28.22	0.97	0.98
Cairo	18.8	2	5.35	13.50	0.64	0.66
Delhi	25.7	3	8.14	18.67	0.90	0.82
Mumbai	21.04	2	5.68	9.00	0.70	0.64

Table S2. The annual total GPP (TgC year^{-1}) from 2000 to 2014 in the world's ten most populous megacities.

Years	São Paulo	Mexico City	Tokyo	Osaka	Beijing	Shanghai	New York–Newark	Cairo	Delhi	Mumbai	Sum
2000	20.99	12.59	20.53	5.35	6.99	7.78	29.41	4.55	9.61	5.75	123.55
2001	23.31	12.59	20.55	5.28	7.23	7.98	33.44	4.62	10.16	5.38	130.55
2002	23.52	12.96	22.59	5.86	7.23	7.71	32.42	4.85	9.16	5.55	131.85
2003	22.38	14.18	21.73	5.43	7.43	7.58	27.16	4.75	10.77	5.31	126.72
2004	23.32	14.70	25.09	6.05	7.99	8.22	32.59	4.89	11.00	5.68	139.53
2005	23.54	13.00	22.43	6.01	8.05	7.83	29.98	4.99	10.16	5.40	131.41
2006	23.46	13.29	22.49	5.52	7.29	7.23	30.54	5.09	10.22	5.54	130.67
2007	23.67	13.84	25.37	6.19	8.29	7.91	33.42	5.03	10.71	5.43	139.85
2008	22.13	12.96	23.76	6.29	8.82	6.84	31.52	4.86	10.88	5.03	133.09
2009	24.02	13.35	25.19	6.44	8.24	6.75	29.63	4.69	10.07	5.43	133.81
2010	22.89	14.56	24.57	6.17	7.38	6.58	34.55	4.42	9.73	5.57	136.42
2011	22.43	12.63	24.71	5.77	7.86	6.26	32.78	4.52	11.07	5.41	133.43
2012	24.27	13.46	25.09	6.34	8.25	7.14	33.83	4.59	9.63	5.35	137.96
2013	22.83	12.79	25.91	6.26	8.04	7.33	32.47	4.76	11.64	5.24	137.25
2014	25.02	13.63	25.05	6.40	7.99	6.28	31.68	4.68	10.53	5.60	136.85
Mean	23.19	13.37	23.67	5.96	7.81	7.29	31.69	4.75	10.36	5.44	133.53
Median	23.32	13.29	24.57	6.05	7.99	7.33	32.42	4.75	10.22	5.43	
Standard deviation	0.97	0.69	1.80	0.40	0.52	0.63	2.00	0.20	0.67	0.18	

Table S3. The linear trends and relationships between annual GPP and GPP_{max}, EVI, and CUP during 2000 – 2014 in the ten megacities. The "--" value of CUP means the vegetation can keep growing over the entire year. "*" indicates a significance level of 0.1.

Megacity name	Trend				Correlation coefficient		
	GPP	GPP_{max}	CUP	EVI	GPP and GPP_{max}	GPP and CUP	GPP and EVI
São Paulo	8.70	0.05	0.00	0.0012	0.71	--	0.53
Mexico City	1.43	-0.02	0.00	0.0005	0.23	--	0.74
Tokyo	16.64*	0.04	1.30	0.0005	0.77	0.78	0.28
Osaka	13.33*	0.04	1.48	0.0028	0.82	0.69	0.68
Beijing	9.44*	0.03	0.57	0.0009	0.58	0.67	0.81
Shanghai	-13.56*	-0.17	0.15	-0.0006	0.90	0.28	0.36
New York–Newark	7.75	0.01	0.71	0.0000	0.69	0.55	0.82
Cairo	-1.83	0.00	0.00	-0.0003	0.79	--	0.81
Delhi	6.78	0.02	0.00	0.0005	0.76	--	0.83
Mumbai	-2.05	-0.04	0.00	0.0005	0.36	--	0.34

Table S4. The linear trends and relationships between annual GPP and population, and nighttime lights during 2000 – 2014 in the ten megacities.

Megacity name	Trend			Correlation coefficient	
	GPP	Population	Nighttime lights	GPP and population	GPP and nighttime lights
São Paulo	8.70	0.27	0.61	0.70	0.05
Mexico City	1.43	0.17	0.40	0.95	-0.10
Tokyo	16.64	0.24	0.23	1.00	0.31
Osaka	13.33	0.11	0.29	0.73	0.46
Beijing	9.44	0.68	1.16	0.30	0.41
Shanghai	-13.56	0.65	1.47	-0.87	-0.71
New York–Newark	7.75	0.05	0.11	0.91	0.44
Cairo	-1.83	0.34	0.89	-0.26	-0.49
Delhi	6.78	0.66	0.98	0.17	0.13
Mumbai	-2.05	0.31	0.51	-0.52	-0.19
